# Comparison of 3 Paclitaxel-Based Chemoradiotherapy Regimens for Patients With Locally Advanced Esophageal Squamous Cell Cancer: A Randomized Clinical Trial

**DOI:** 10.1001/jamanetworkopen.2022.0120

**Published:** 2022-02-21

**Authors:** Dashan Ai, Jinjun Ye, Shihong Wei, Yunhai Li, Hui Luo, Jianzhong Cao, Zhengfei Zhu, Weixin Zhao, Qin Lin, Huanjun Yang, Xiangpeng Zheng, Jialiang Zhou, Guang Huang, Ling Li, Jiancheng Li, Zhi Zhang, Guoren Zhou, Dayong Gu, Mingyu Du, Miao Mo, HuiXun Jia, Zhen Zhang, Kuaile Zhao

**Affiliations:** 1 Department of Radiation Oncology, Fudan University Shanghai Cancer Center, Shanghai, China; 2 Department of Oncology, Shanghai Medical College, Fudan University, Shanghai, China; 3 Department of Radiation Oncology, Jiangsu Cancer Hospital, Jiangsu Institute Of Cancer Research, Affiliated Cancer Hospital of Nanjing Medical University, Nanjing, China; 4 Department of Radiation Oncology, Gansu Province Cancer Hospital, Lanzhou, China; 5 Department of Radiation Oncology, Fudan University Shanghai Cancer Center Minhang Branch Hospital, Shanghai, China; 6 Department of Radiation Oncology, Jiangxi Province Cancer Hospital, Nanchang, China; 7 Department of Radiation Oncology, Shanxi Province Cancer Hospital, Taiyuan, China; 8 Department of Radiation Oncology, First Affiliated Hospital of Xiamen University, Xiamen, China; 9 Department of Radiation Oncology, Huadong Hospital Affiliated to Fudan University, Shanghai, China; 10 Department of Radiation Oncology, Affiliated Hospital of Jiangnan University, Wuxi, China; 11 Department of Radiation Oncology, Hainan Province People’s Hospital, Haikou, China; 12 Department of Radiation Oncology, Fujian Province Cancer Hospital, Fuzhou, China; 13 Department of Thoracic Surgery, Jiangsu Cancer Hospital, Jiangsu Institute Of Cancer Research, Affiliated Cancer Hospital of Nanjing Medical University, Nanjing, China; 14 Department of Internal Medicine, Jiangsu Cancer Hospital, Jiangsu Institute Of Cancer Research, Affiliated Cancer Hospital of Nanjing Medical University, Nanjing, China; 15 Department of Cancer Prevention & Clinical Statistics Center, Fudan University Shanghai Cancer Center, Shanghai, China; 16 Shanghai General Hospital, Shanghai, China

## Abstract

**Importance:**

Multiple paclitaxel-based regimens are widely used in chemoradiation therapy against esophageal cancer, including regimens combining paclitaxel with fluorouracil, cisplatin, and carboplatin. However, which among these 3 regimens provides the best prognosis with minimum adverse events is still unknown.

**Objective:**

To compare the efficacy and adverse events of fluorouracil, cisplatin, and carboplatin in definitive chemoradiotherapy in patients with esophageal squamous cell carcinoma (ESCC).

**Design, Setting, and Participants:**

This randomized clinical trial of patients with ESCC was conducted in 11 treatment centers in China. Eligible patients were aged 18 to 75 years and had histologically confirmed ESCC stages IIa to IVa with no prior treatment, Eastern Cooperative Oncology Group performance status of 2 or lower, and adequate organ functions. The study was conducted between July 2015 and February 2018, and the cutoff date for data analysis was August 31, 2020.

**Interventions:**

Patients with locally advanced ESCC were randomly assigned (1:1:1) to groups combining paclitaxel treatment with fluorouracil, cisplatin, or carboplatin. Patients in the cisplatin group were treated with 2 cycles of concurrent chemoradiotherapy followed by 2 cycles of consolidation chemotherapy with monthly paclitaxel plus cisplatin. For the fluorouracil group, patients were administered 6 cycles of weekly paclitaxel plus fluorouracil in concurrent chemoradiotherapy followed by 2 cycles of monthly paclitaxel plus fluorouracil in consolidation chemotherapy. Patients in the carboplatin group were treated with 6 cycles of weekly paclitaxel plus carboplatin in concurrent chemoradiotherapy followed by 2 cycles of monthly paclitaxel plus carboplatin in consolidation chemotherapy. All patients received radiotherapy of 61.2 Gy delivered in 34 fractions.

**Main Outcomes and Measures:**

The primary end point was overall survival (OS). The secondary end points were progression-free survival and adverse events.

**Results:**

Overall, 321 patients (median [IQR] age, 64 years [59-69 years]; 248 [77.3%] men) with ESCC from 11 centers were randomized into fluorouracil, cisplatin, or carboplatin groups between July 2015 and February 2018. Over a median (IQR) follow-up time of surviving patients of 46.0 months (36.6-53.0 months), the 3-year OS rates were 57.2% in the fluorouracil group, 60.1% in the cisplatin group, and 56.5% in the carboplatin group, respectively (fluorouracil vs cisplatin: HR, 1.06; 95% CI, 0.71-1.60; *P* = .77; fluorouracil vs carboplatin: HR, 0.94; 95% CI, 0.63-1.40; *P* = .77). The cisplatin group had significantly higher incidences of acute grade 3 or 4 neutropenia (69 events [60.8%] vs 19 [17.8%] for fluorouracil and 37 [34.6%] carboplatin; *P* < .001), thrombocytopenia (14 events [13.1%] vs 4 [3.7%] for fluorouracil and 5 [4.7%] for carboplatin; *P* = .01), anemia (50 events above grade 2 [46.7%] vs 25 [23.4%] for fluorouracil and 37 [34.6%] for carboplatin; *P* = .35), fatigue (11 events [10.3%] vs 2 [1.9%] for fluorouracil and 1 [0.9%] carboplatin; *P* = .007), and vomiting (17 events above grade 2 [15.9%] vs 3 [2.8%] for fluorouracil and 5 [4.7%] for carboplatin; *P* < .001) than the other 2 groups.

**Conclusions and Relevance:**

In this randomized clinical trial, paclitaxel plus fluorouracil did not show OS superiority over paclitaxel plus cisplatin or paclitaxel plus carboplatin regimens in definitive chemoradiation in patients with locally advanced ESCC. Higher rates of hematologic and gastrointestinal toxic effects were reported in the cisplatin group compared with those in the fluorouracil or carboplatin groups.

**Trial Registration:**

ClinicalTrials.gov Identifier: NCT02459457

## Key Points

**Question:**

Which paclitaxel-based regimen, among fluorouracil, cisplatin, and carboplatin, provides the best prognosis with minimum adverse events for patients with locally advanced esophageal squamous cell carcinoma?

**Findings:**

In this randomized clinical trial of 321 patients with esophageal squamous cell carcinoma treated in 11 cancer centers in China, the 3-year overall survival rates were 57.2% in the fluorouracil group, 60.1% in the cisplatin group, and 56.5% in the carboplatin group, respectively. The paclitaxel plus fluorouracil regimen did not show overall survival superiority over paclitaxel regimens with cisplatin or carboplatin in patients with locally advanced esophageal squamous cell carcinoma who are in chemoradiation therapy.

**Meaning:**

Because different regimens may lead to different adverse effects in patients treated with concurrent chemoradiotherapy for locally advanced esophageal cancer, these results suggest that specialists have more choices in clinical practice and for future research that can account for the needs of patients, without sacrificing odds of survival.

## Teaser

This randomized clinical trial compares 3-year overall survival rates and adverse events for chemoradiotherapy using paclitaxel in combination with fluorouracil, cisplatin, or carboplatin among patients with esophageal squamous cell carcinoma.

## Introduction

Esophageal cancer is the third most common cancer in China and was responsible for an estimated 477 900 new cases and 375 000 deaths in 2015.^1^ Most of the cases are squamous cell carcinoma. Concurrent chemoradiation is the standard nonoperative therapy for locally advanced esophageal squamous cell carcinoma (ESCC).^2^

Paclitaxel is an active therapeutic agent against esophageal cancer, and it has been proven to be a radiation sensitizer. Previously, our group conducted the ESO-Shanghai 1 trial,^3^ a multicenter randomized phase III trial showing that paclitaxel-based chemotherapy concurrent with radiotherapy led to similar 3-year overall survival (OS) compared with a traditional cisplatin plus fluorouracil regimen (55.4% vs 51.8%). In the comments from Rogers and Ajani on this study,^4^ cisplatin was identified as an inherently gastrointestinal toxic agent compared with a paclitaxel-based chemotherapy. These toxic effects are extremely detrimental to patients with esophageal cancer because of high incidence rates of weight loss and malnutrition. Therefore, a trend toward abandoning cisplatin-based regimens and embracing a paclitaxel-based regimen has been noted, and the National Comprehensive Cancer Network (NCCN) also downgraded the use of cisplatin and listed paclitaxel-based regimens as alternatives.

In clinical practice, multiple paclitaxel-based regimens are widely used in chemoradiation therapy against esophageal cancer. The paclitaxel plus fluorouracil regimen was developed at the University of Texas MD Anderson Cancer Center; it includes 300 mg/m^2^ of fluorouracil and 50 mg/m^2^ of paclitaxel per week concurrent with radiation therapy.^5^ The paclitaxel plus cisplatin regimen, originated by the Cleveland Clinic Foundation, includes 20 mg/m^2^ of cisplatin for 4 days and 175 mg/m^2^ of paclitaxel over 24 hours every 3 weeks concurrent with radiation therapy.^6^ The RTOG 0113 trial,^5^ a phase II trial, compared fluorouracil-based therapy and non-fluorouracil–based therapy (ie, the cisplatin group in this study), and the median survival time of patients was 28.7 months in the fluorouracil group and 14.9 months in the cisplatin group, respectively. Another plan from the Chemoradiotherapy for Oesophageal Cancer Followed by Surgery Study (CROSS) trial^7^ was the paclitaxel plus carboplatin plan, which had a carboplatin targeted at an area under the curve of 2 mg/mL/min and a 50 mg/m^2^ dose of paclitaxel administered weekly concurrent with radiation therapy. Given the efficacy and safety of the carboplatin plan as a preoperative chemoradiation regimen, this regimen was recommended as a preferred option for definitive chemoradiation in the NCCN guideline, even without a large-scale clinical trial testing it as definitive chemoradiation therapy. When examined in retrospective studies, the carboplatin regimen was reported to have an OS of 13.8 to 17.4 months in definitive chemoradiation. Seemingly, the fluorouracil plan showed the best prognosis among these 3 regimens. However, which of these 3 paclitaxel-based regimens provides the best prognosis in combination with minimum adverse events is still unknown, and very few large-scale studies focus on this topic.

Initiated in July 2015, this trial (ESO-Shanghai 2; ClinicalTrials.gov Identifier, NCT02459457) was a 3-arm, multicenter, open-labeled, randomized phase III clinical trial to confirm the superiority of paclitaxel plus fluorouracil to paclitaxel regimens with cisplatin or carboplatin concurrent with definitive radiotherapy in terms of improving overall survival for patients with locally advanced ESCC.

## Methods

### Study Design

Eleven centers around China participated in the ESO-Shanghai 2 trial (eTable 1 in Supplement 1). A detailed description of the protocol for the study has previously been published.^8^ The protocol was approved by the ethics committee of the Fudan University Shanghai Cancer Center and is available in Supplement 2. All participants provided written informed consent. This study followed the Consolidated Standards of Reporting Trials (CONSORT) reporting guideline for randomized clinical trials.

### Inclusion Criteria and Group Assignment

Patients participating in this study met the following key eligibility criteria: histologically confirmed esophageal squamous cell carcinoma, stage IIa to IVa disease (based on the American Joint Committee on Cancer [AJCC] *Cancer Staging Manual, 6th edition* guidelines); no prior treatment; aged 18 to 75 years old; Eastern Cooperative Oncology Group performance status of 2 or lower; no severe abnormalities in hematopoietic, cardiac, pulmonary, kidney, or hepatic function; and adequate hematologic function (eTable 2 in Supplement 1).

After the confirmation of eligibility criteria, patients were randomly allocated in a 1:1:1 ratio to 1 of the 3 treatment groups (paclitaxel plus fluorouracil, cisplatin, or carboplatin) by a central randomization center (Fudan University Shanghai Cancer Center). Patients were stratified by lymph node status (N0, N1, and M1a). SAS software version 9.0 (SAS Institute) was used to generate a random permutation sequence and produce patient randomization numbers. The data center registered the enrollment, assigned a unique identification number to every participant, and responded to the respective investigators. Treatment assignment was not masked to participants or physicians. Patient age and sex were tracked by the study; race and ethnicity were not.

### Treatment

All assigned patients were scheduled to receive concurrent chemoradiotherapy followed by consolidation chemotherapy. The first fraction of radiotherapy and the first cycle of chemotherapy started on the same day. Patients in all 3 groups received the same radiotherapy regimen with photons (≥6 mV) to a total dose of 61.2 Gy (to convert gray to rads, multiply by 100) in 34 fractions (5 days per week at 1.8 Gy/d) to planning target volume. The gross target volume was defined as all known involved fields. The superior and inferior borders of the clinical target volume were 3 cm beyond the primary tumor along the esophagus. The lateral, anterior, and posterior borders of the field were the same as the gross target volume. All borders of the planning target volume were 1 cm beyond the clinical target volume. Intensity modulated radiotherapy was required for all patients.

Patients in the cisplatin group were treated with 2 cycles of concurrent chemoradiotherapy followed by 2 cycles of consolidation chemotherapy with paclitaxel plus cisplatin (cisplatin, 25 mg/m^2^/d, days 1-3; paclitaxel, 175 mg/m^2^, day 1, per 28 days). The fluorouracil group received 6 cycles paclitaxel plus fluorouracil (fluorouracil, 300 mg/m^2^, civ 96h; paclitaxel, 50 mg/m^2^, day 1, per week) in concurrent chemoradiotherapy followed by 2 cycles (fluorouracil, 1800 mg/m^2^, continuous IV infusion 72 hours; paclitaxel, 175 mg/m^2^, day 1, per 28 days) in consolidation chemotherapy. Patients in the carboplatin group were treated with 6 cycles of paclitaxel plus carboplatin (carboplatin, area under the curve, 2, day 1; paclitaxel, 50 mg/m^2^, day 1, per week) in concurrent chemoradiotherapy followed by 2 cycles (carboplatin, area under the curve, 5, day 1; paclitaxel, 175 mg/m^2^, day 1, per 28 days) in consolidation chemotherapy.

### Outcomes

The primary end point was OS of all randomized patients. We defined OS as the time from the date of randomization until death from any cause or the last follow-up for patients alive at the end of study. The secondary end points were progression-free survival (PFS) and adverse events. PFS was defined as the time from the date of randomization to the date of progression or the date of death, whichever occurred first. Disease progression was evaluated at months 3, 6, 9, 12, 15, 18, 21, 24, 30, 36, 42, 48, 54, and 60 after last treatment according to Responsive Evaluation Criteria in Solid Tumours (RECIST) version 1.1 criteria. Chest computed tomographic scans with contrast, abdomen ultrasounds, and barium swallows were to be processed routinely with esophagoscope when necessary. Adverse events were evaluated according to the Common Terminology Criteria for Adverse Events (CTCAE) version 4.0 (National Cancer Institute) at the same frequency for all 3 groups.

### Statistical Analysis

According to the RTOG 0113 study and other retrospective reports, the median survival time of patients with ESCC receiving paclitaxel plus fluorouracil concurrent with radiotherapy is 28.7 months,^5^ compared with 14.9 months for paclitaxel plus cisplatin^5^ and 17.4 months for paclitaxel plus carboplatin.^9^ Based on these data, a minimum sample size of 321 patients (107 patients per arm) would be necessary to warrant a power of 80% at a 2-sided α level of .025 to demonstrate the superiority of fluorouracil to the cisplatin or carboplatin groups, assuming an accrual period of 48 months, a minimum follow-up period of 24 months, and a dropout rate of 5%. The Kaplan-Meier method was used to estimate the event time and an unadjusted log-rank test was used to compare the OS and PFS rates among patients in the treatment arms. Cox regression analysis was performed to estimate the hazard ratios of overall survival of the fluorouracil vs the cisplatin and carboplatin groups. Pearson χ^2^ or Fisher exact tests were used to compare the toxic effects and treatment completion rates between patients in different groups. The significance threshold for the analysis was set as 2-sided *P* < .025 for the primary end point. Data were analyzed with SPSS version 19.0 (IBM Corp).

## Results

**Figure 1.**
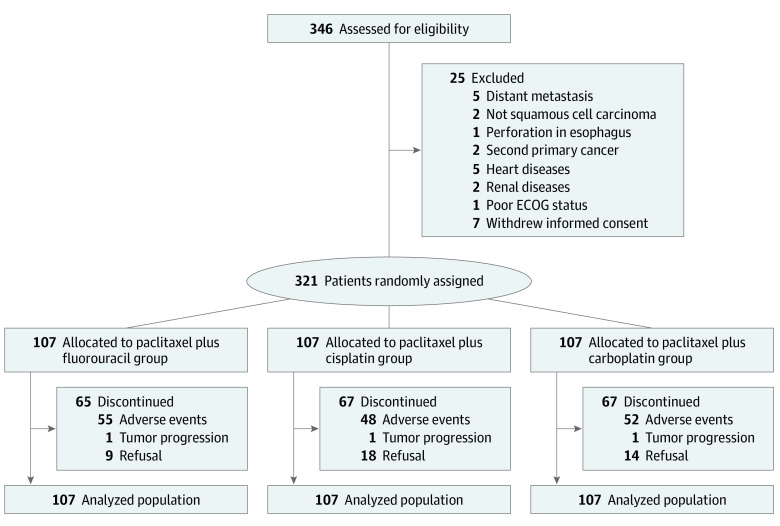
Trial Profile ECOG indicates Eastern Cooperative Oncology Group. Three hundred forty-six patients with esophageal squamous cell carcinoma were assessed for eligibility at registration in 11 centers in China. Three hundred twenty-one patients were randomly assigned to the paclitaxel plus fluorouracil group, the paclitaxel plus cisplatin group, or the paclitaxel plus carboplatin group as an intention-to-treat population.

Between July 1, 2015, and February 26, 2018, a total of 321 patients with esophageal cancer from 11 centers were randomized into the fluorouracil, cisplatin, or carboplatin groups (eTable 1 in Supplement 1; Figure 1). Baseline characteristics were well balanced among the 3 groups. The median (IQR) age was 63.0 (59-67), 65.0 (59-69), and 64.0 (60-68) years in the fluorouracil, cisplatin, and carboplatin groups, respectively. Because we restaged for supraclavicular lymph node metastasis after reviewing the pretreatment radiology examinations, 23 patients (10 patients [9.3%] in the fluorouracil group, 9 patients [8.4%] in the cisplatin group, and 4 patients [3.7%] in the carboplatin group) were upstaged to IVb. The median (IQR) tumor lengths of the patients in the fluorouracil, cisplatin, and carboplatin groups were 5.0 cm (3.0-6.0 cm), 5.0 cm (3.0-6.0 cm), and 5.0 cm (4.0-7.0 cm), respectively (Table 1).

**Table 1.** Patient Characteristics

Characteristics	Patients, No. (%)		
	Paclitaxel plus fluorouracil (n = 107)	Paclitaxel plus cisplatin (n = 107)	Paclitaxel plus carboplatin (n = 107)
Age, y			
≤65	66 (61.7)	58 (54.2)	63 (58.9)
>65	41 (38.3)	49 (45.8)	44 (41.1)
Sex			
Men	80 (74.8)	82 (76.6)	86 (80.4)
Women	27 (25.2)	25 (23.4)	21 (19.6)
ECOG performance status			
0	54 (50.5)	50 (46.7)	47 (43.7)
1	53 (49.5)	57 (53.3)	60 (56.1)
Stage at diagnosis^a^			
IIa	13 (12.1)	16 (15.0)	12 (11.2)
IIb	15 (14.0)	10 (9.3)	12 (11.2)
III	47 (43.9)	55 (51.4)	66 (61.7)
IVa	22 (20.6)	17 (15.9)	13 (12.1)
IVb^b^	10 (9.3)	9 (8.4)	4 (3.7)
Reason for no surgery			
Local extent of disease	77 (72.0)	69 (64.5)	68 (63.6)
Surgical contraindication	20 (18.7)	21 (19.6)	25 (23.4)
Patient refusal	10 (9.3)	17 (15.9)	14 (13.1)
Tumor length, cm			
≤5	66 (61.7)	70 (65.4)	68 (63.6)
>5	41 (38.3)	37 (34.6)	39 (36.4)
Tumor location			
Cervical	12 (11.2)	16 (15.0)	17 (15.9)
Upper thoracic	58 (54.2)	39 (36.4)	49 (45.8)
Middle thoracic	28 (26.2)	39 (36.4)	36 (33.6)
Lower thoracic	6 (5.6)	11 (10.3)	3 (2.8)
Multiple	3 (2.8)	2 (1.9)	2 (1.9)

Abbreviation: ECOG, Eastern Cooperative Oncology Group.

^a^ Based on the American Joint Committee on Cancer’s *Cancer Staging Manual, 6th edition* staging.

^b^ A total of 23 patients (10 patients [9.3%] in the paclitaxel plus fluorouracil group, 9 patients [8.4%] in the paclitaxel plus cisplatin group, and 4 patients [3.7%] in the paclitaxel plus carboplatin group) were upstaged from IVa to IVb after staging review.

Chemotherapy compliance details in randomly assigned patients are listed in eTable 3 in Supplement 1. More than 75% of randomized patients (81 patients [75.7%] in the fluorouracil group, 94 patients [87.9%] in the cisplatin group, and 85 patients [79.4%] in the carboplatin group) completed at least 80% of the concurrent chemoradiotherapy per protocol. A greater number of the patients in the cisplatin group (38 patients [35.5%]) required dose modifications than those in the fluorouracil (6 patients [5.6%]) or carboplatin (9 patients [8.4%]) groups.

Details of radiotherapy compliance in the 3 groups are shown in eTable 4 in Supplement 1. A high completion rate of radiotherapy was observed in all 3 groups (93.5% in the fluorouracil group, 95.3% in the cisplatin group, and 89.7% in the carboplatin group). However, treatment-induced toxic effects led to more interruptions in the cisplatin group (41 patients [38.3%]) than in the carboplatin (28 patients [26.2%]) or the fluorouracil (25 patients [23.4%]) group.

**Figure 2.**
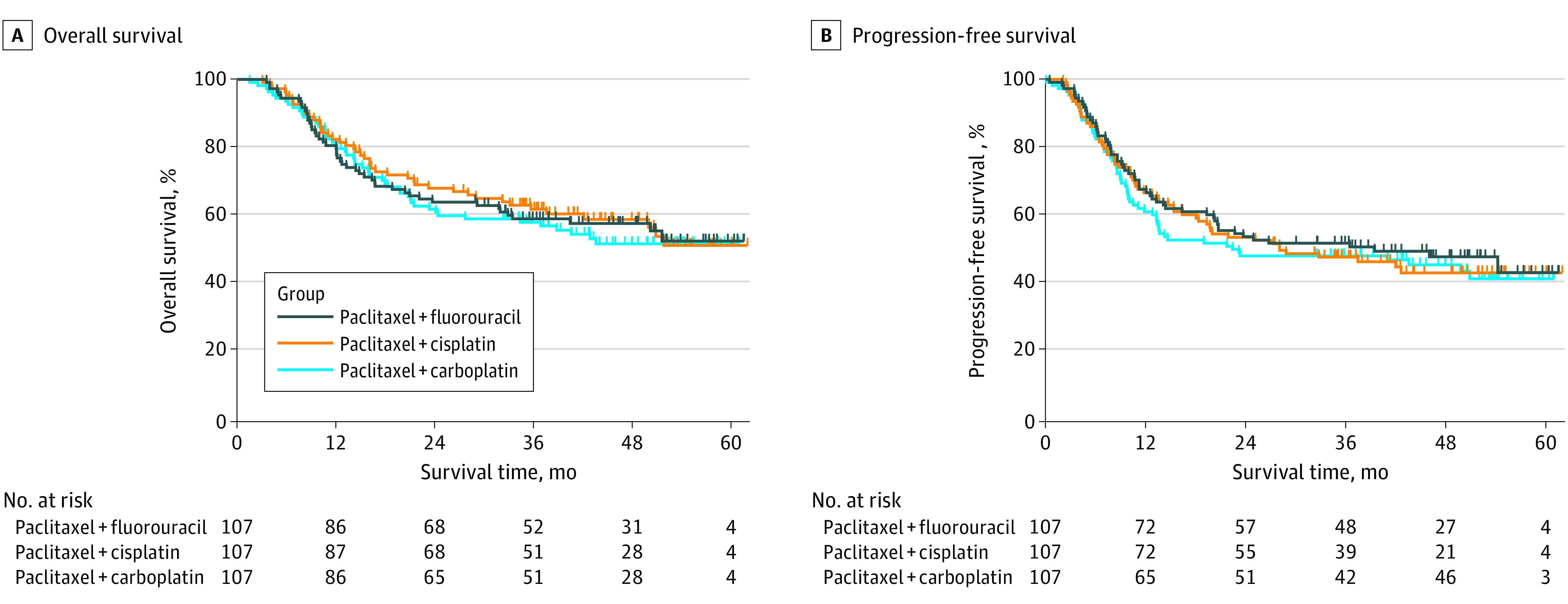
Overall Survival and Progression-Free Survival in Enrolled Patients

At the time of analysis on August 31, 2020, the median (IQR) follow-up time was 46.0 months (36.6-53.0 months). One hundred forty-two deaths (44.2%) were recorded, including 47 (43.9%) in the fluorouracil group, 45 (42.1%) in the cisplatin group, and 50 (46.7%) in the carboplatin group. Median (IQR) overall survival was not reached in all 3 groups, respectively. The 1-, 2-, and 3-year OS rates were 79.4% (71.8%-87.0%), 62.6% (53.4%-71.8%), and 57.2% (47.6%-66.8%), respectively, for the fluorouracil group; 81.3% (73.9%-88.7%), 66.7% (57.7%-75.7%), and 60.1% (50.5%-69.7%), respectively, for the cisplatin group; and 79.4% (71.8%-87.0%), 60.5% (51.3%-69.7%), and 56.5% (47.1%-65.9%), respectively, for the carboplatin group. Fluorouracil did not show OS superiority over the cisplatin or carboplatin regimens in chemoradiation therapy in patients with locally advanced ESCC (fluorouracil vs cisplatin: HR, 1.06; 95% CI, 0.71-1.60; *P* = .77; fluorouracil vs carboplatin: HR, 0.94; 95% CI, 0.63-1.40; *P* = .77) (Figure 2). These results were consistent considering relevant prognostic factors (eFigure 1 and 2 in Supplement 1).

One hundred forty-six patients (46.1%) were alive without disease progression at the time of analysis on August 31, 2020, including 51 (47.6%) in the fluorouracil group, 48 (44.9%) in the cisplatin group, and 47 (43.9%) in the carboplatin group. The 1-, 2-, and 3-year PFS rates were 66.4% (57.4%-75.4%), 52.3% (42.9%-61.7%), and 50.3% (40.7%-59.9%), respectively, for the fluorouracil group, 65.4% (56.4%-74.4%), 52.2% (42.8%-61.6%), and 45.9% (36.3%-55.5%), respectively, for the cisplatin group, and 59.8% (50.6%-69.0%), 46.4% (36.8%-56.0%), and 46.4% (36.8%-56.0%), respectively, for the carboplatin group (Figure 2). Moreover, no superiority in locoregional recurrence-free survival and distant metastasis-free survival were observed among the 3 groups (eFigure 3 in Supplement 1). Patterns of treatment failure are shown in eTable 5 in Supplement 1.

All grade 3 or higher acute events and grade 1 to 2 acute events with an incidence of more than 10% are listed in Table 2. The cisplatin group showed higher incidence rates of acute grade 3 or 4 hematologic toxic effects, such as neutropenia (69 events [60.8%] vs 19 [17.8%] for fluorouracil and 37 [34.6%] carboplatin; *P* < .001), thrombocytopenia (14 events [13.1%] vs 4 [3.7%] for fluorouracil and 5 [4.7%] for carboplatin; *P* = .01), and gastrointestinal toxic effects, such as grade 2 or higher vomiting (17 events [15.9%] vs 3 [2.8%] for fluorouracil and 5 [4.7%] for carboplatin; *P* < .001), than the other 2 groups. The fluorouracil and carboplatin group exhibited significantly higher incidence rates than the cisplatin group of grade 2 or higher esophagitis (27.1% [29 events] for cisplatin vs 43.0% [46 events] for fluorouracil and 41.1% [44 events] for carboplatin; *P* = .03) and pneumonitis (4.7% [5 events] vs 26.2% [28 events] for fluorouracil and 21.5% [22 events] for carboplatin; *P* < .001). One patient in the fluorouracil group and 1 in the carboplatin group died of acute esophagitis. One patient in the fluorouracil group died of acute pneumonitis. A higher proportion of patients with late esophagitis were observed in the fluorouracil group than in the cisplatin or carboplatin groups (17 events [15.9%] vs 8 [7.5%] for cisplatin and 6 [5.6%] for carboplatin; *P* = .03), while no differences in late cardiac events or pneumonitis were observed (Table 3).

**Table 2.** Acute Adverse Events of Patients by Treatment Group

Adverse event^a^	Events, No. (%)															P value^b^
	Paclitaxel plus fluorouracil (n = 107)					Paclitaxel plus cisplatin (n = 107)					Paclitaxel plus carboplatin (n = 107)					
	Grade 1	Grade 2	Grade 3	Grade 4	Grade 5	Grade 1	Grade 2	Grade 3	Grade 4	Grade 5	Grade 1	Grade 2	Grade 3	Grade 4	Grade 5	
Hematological																
Anemia	62 (57.9)	23 (21.5)	2 (1.9)	0	0	46 (43.0)	44 (41.1)	6 (5.6)	0	0	66 (61.7)	33 (30.8)	4 (3.7)	0	0	.35
Leukopenia	18 (16.8)	36 (33.6)	27 (25.2)	6 (5.6)	0	4 (3.7)	22 (20.6)	36 (33.6)	33 (30.8)	0	10 (9.3)	31 (29.0)	53 (49.5)	7 (6.5)	0	<.001
Neutropenia	15 (14.0)	27 (25.2)	11 (10.3)	8 (7.5)	0	7 (6.5)	15 (14.0)	19 (17.8)	46 (43.0)	0	18 (16.8)	33 (30.8)	21 (19.6)	16 (15.0)	0	<.001
Thrombocytopenia	22 (20.6)	5 (4.7)	4 (3.7)	0	0	40 (37.4)	33 (30.8)	12 (11.2)	2 (1.9)	0	31 (29.0)	17 (15.9)	5 (4.7)	0	0	.01
Gastrointestinal																
Anorexia	31 (29.0)	16 (15.0)	1 (0.9)	0	0	32 (29.9)	30 (28.0)	1 (0.9)	0	0	24 (22.4)	20 (18.7)	1 (0.9)	0	0	.06
Nausea	30 (28.0)	13 (12.1)	0	0	0	25 (23.4)	30 (28.0)	2 (1.9)	0	0	27 (25.2)	13 (12.1)	0	0	0	.001
Vomiting	11 (10.3)	3 (2.8)	0	0	0	11 (10.3)	12 (11.2)	5 (4.6)	0	0	12 (11.2)	4 (3.7)	1 (0.9)	0	0	<.001
Diarrhea	2 (1.9)	0	0	0	0	8 (7.5)	4 (3.7)	0	1 (0.9)	0	3 (2.8)	0	1 (0.9)	0	0	.03
Constipation	24 (22.4)	7 (6.5)	0	0	0	34 (31.8)	6 (5.6)	0	0	0	24 (22.4)	6 (5.6)	0	0	0	.95
Constitutional symptoms																
Fatigue	42 (39.3)	14 (13.1)	2 (1.9)	NA	NA	46 (43.0)	15 (14.0)	11 (10.3)	NA	NA	38 (35.5)	19 (17.8)	1 (0.9)	NA	NA	.007
Renal and hepatic																
ALT increased	9 (8.4)	2 (1.9)	0	0	NA	7 (6.5)	4 (3.7)	0	0	NA	9 (8.4)	0	0	0	NA	.13
AST increased	8 (7.5)	0	0	0	NA	7 (6.5)	1 (0.9)	0	0	NA	6 (5.6)	0	0	0	NA	.37
Total bilirubin increased	11 (10.3)	6 (5.6)	0	0	NA	13 (12.1)	5 (4.7)	0	0	NA	13 (12.1)	5 (4.7)	0	0	NA	.94
Creatinine increased	2 (1.9)	1 (0.9)	0	0	NA	7 (6.5)	1 (0.9)	0	0	NA	8 (7.5)	1 (0.9)	0	0	NA	>.99
Nutrition																
Hypoalbuminemia	55 (51.4)	5 (4.7)	0	0	0	42 (39.3)	6 (5.6)	0	0	0	46 (43.0)	5 (4.7)	0	0	0	.94
Hypokalemia	12 (11.2)	1 (0.9)	1 (0.9)	0	0	17 (15.9)	7 (6.5)	4 (3.7)	0	0	11 (10.3)	4 (3.7)	2 (1.9)	0	0	.03
Hyponatremia	21 (19.6)	NA	0	0	0	28 (26.2)	NA	3 (2.8)	3 (2.8)	0	31 (29.0)	NA	2 (1.9)	0	0	.03
Mediastinal																
Hiccup	25 (23.4)	3 (2.8)	0	NA	NA	31 (29.0)	9 (8.4)	0	NA	NA	29 (27.1)	3 (2.8)	2 (1.9)	NA	NA	.18
Hoarseness	27 (25.2)	7 (6.5)	0	NA	NA	26 (24.3)	10 (9.3)	0	NA	NA	29 (27.1)	7 (6.5)	1 (0.9)	NA	NA	.74
Neurological																
Dizziness	24 (22.4)	2 (1.9)	0	NA	NA	29 (27.1)	9 (8.4)	0	NA	NA	31 (29.0)	2 (1.9)	0	NA	NA	.02
Headache	19 (17.8)	0	0	NA	NA	14 (13.1)	5 (4.7)	0	NA	NA	14 (13.1)	2 (1.9)	0	NA	NA	.06
Arthralgia and myalgia	30 (28.0)	5 (4.7)	0	NA	NA	29 (27.1)	13 (12.1)	1 (0.9)	NA	NA	24 (22.4)	4 (3.7)	0	NA	NA	.01
Peripheral neuropathy	23 (21.5)	5 (4.7)	0	0	0	37 (34.6)	11 (10.3)	0	0	0	27 (25.2)	5 (4.7)	1 (0.9)	0	0	.22
Radiation induced																
Dermatitis	47 (43.9)	12 (11.2)	4 (3.7)	0	0	44 (41.1)	7 (6.5)	0	0	0	47 (43.9)	17 (15.9)	2 (1.9)	0	0	.04
Esophagitis	53 (49.5)	34 (31.8)	9 (8.4)	2 (1.9)	1 (0.9)	69 (64.5)	28 (26.2)	0	1 (0.9)	0	56 (52.3)	38 (35.5)	5 (4.7)	0	1 (0.9)	.03
Pneumonitis	32 (29.9)	23 (21.5)	4 (3.7)	0	1 (0.9)	44 (41.1)	5 (4.7)	0	0	0	34 (31.8)	21 (19.6)	2 (1.9)	0	0	<.001
Hair loss	22 (20.6)	9 (8.4)	NA	NA	NA	20 (18.7)	40 (37.4)	NA	NA	NA	29 (27.1)	11 (10.3)	NA	NA	NA	<.001
Cardiac	9 (8.4)	2 (1.9)	0	0	0	15 (14.0)	1 (0.9)	0	0	0	11 (10.3)	0	0	0	0	.36

Abbreviations: ALT, alanine aminotransferase; AST, aspartate aminotransferase; NA, not applicable.

^a^ All grade 3 or higher acute adverse events and grade 1 to 2 acute adverse events occurring in over 10% of patients that were reported during treatment.

^b^ *P* values compared grade 3 or higher hematologic adverse events and grade 2 or higher nonhematologic adverse events between groups.

**Table 3.** Late Adverse Events of Patients by Treatment Group

Adverse event	Events, No. (%)															P value^a^
	Paclitaxel plus fluorouracil (n = 107)					Paclitaxel plus cisplatin (n = 107)					Paclitaxel plus carboplatin (n = 107)					
	Grade 1	Grade 2	Grade 3	Grade 4	Grade 5	Grade 1	Grade 2	Grade 3	Grade 4	Grade 5	Grade 1	Grade 2	Grade 3	Grade 4	Grade 5	
Cardiac	9 (8.4)	5 (4.7)	0	0	0	9 (8.4)	0	0	0	0	15 (14.0)	1 (0.9)	0	0	0	.32
Esophagitis	8 (7.5)	5 (4.7)	3 (2.8)	1 (0.9)	0	5 (4.7)	2 (1.9)	1 (0.9)	0	0	3 (2.8)	2 (1.9)	1 (0.9)	0	0	.03
Pneumonitis	33 (30.8)	5 (4.7)	0	0	0	29 (27.1)	6 (5.6)	0	0	0	32 (29.9)	2 (1.9)	1 (0.9)	0	0	.88

^a^ *P* values compared all grade late adverse events among 3 groups.

## Discussion

To the best of our knowledge, our study is the first randomized, multicenter, phase III clinical trial comparing the efficacy and safety of various paclitaxel-based dual-drug regimens concurrent with radiotherapy in the treatment of locally advanced esophageal cancer. In our study, no superiority in overall survival was observed between groups treated with fluorouracil vs cisplatin or fluorouracil vs carboplatin, even when accounting for different treatment regimens and frequencies. Higher rates of hematologic and gastrointestinal toxic effects in the cisplatin group compared with the fluorouracil or carboplatin regimens led to more interruptions in radiotherapy.

The protocol of the fluorouracil group in this study was almost the same with that in the ESO-Shanghai 1 study,^3^ and the prognosis of patients in the 2 studies was similar, with 2-year OS rates of 62.6% in this study and 60.6% in ESO-Shanghai 1, results that were also similar to the prognosis of the fluorouracil group in the RTOG 0113 study, which had 2-year OS rates of 56%.^5^ In our study, less severe gastrointestinal adverse events and a lower rate of grade IV neutropenia were observed in the fluorouracil group compared with the carboplatin or cisplatin groups. However, the fluorouracil group had relatively higher incidences of esophagitis and pneumonitis than the cisplatin group, consistent with the RTOG 0113 study (grade 2 or higher pneumonitis: 18.9% in the fluorouracil-based arm vs 11.4% in the non-fluorouracil–based arm).^5^

The carboplatin plan showed excellent efficacy in the CROSS study. The pathological complete response rate of squamous cell carcinoma was as high as 49% after 5 cycles of carboplatin chemotherapy and radiotherapy with a total dose of 41.4 Gy at 1.8 Gy per fraction.^7^ Since then, the carboplatin plan was widely accepted and recommended by NCCN guidelines as the standard chemotherapy regimen for concurrent chemoradiotherapy, including neoadjuvant and definitive therapy. However, in the absence of a prospective comparison, no difference in prognosis was observed between patients receiving paclitaxel plus carboplatin and cisplatin plus fluorouracil in definitive chemoradiotherapy.^10^ Regarding safety, patients in the carboplatin group experienced mild adverse effects, with fewer hematological and gastrointestinal toxic effects than those in the cisplatin group, but still more severe toxic effects than those in the CROSS study, especially hematologic toxic effects (34.6% for over grade 3 neutropenia), which was potentially attributed to more chemotherapy cycles.^7^

The design of the cisplatin plan in this study, a monthly plan, was modeled on the cisplatin regimen from the study of perioperative chemoradiotherapy.^6^ Definitive chemoradiotherapy with a monthly cisplatin regimen is commonly used in China,^11^ which differs from the weekly cisplatin regimen used in the RTOG 0113 study.^5^ In our study, without better prognosis, the cisplatin group had significantly more severe gastrointestinal (grade ≥2 vomiting, 15.9% vs 2.8% for fluorouracil or 4.7% for carboplatin regimes) and hematologic adverse events (grade 4 neutropenia, 43.0% vs 7.5% for fluorouracil or 15.0% carboplatin regimes) than the other 2 groups, resulting in more chemotherapy dose reductions (35.5% vs 5.6% for fluorouracil or 8.4% for carboplatin) and radiotherapy interruptions (38.3% vs 23.4% for fluorouracil or 26.2% for carboplatin).

Traditionally, cisplatin tends to induce more neutropenia and thrombocytopenia than fluorouracil, which was verified in the RTOG 0113 study (grade ≥3 myelotoxicity, 38% in the fluorouracil-based group vs 69% in the cisplatin-based group).^5^ Moreover, the addition of paclitaxel to cisplatin led to more severe hematologic adverse events. In the study by Adelstein et al,^6^ the paclitaxel-based regimen was associated with significantly more neutropenia (grade ≥3 neutropenia, 95% in the paclitaxel-based group vs 43% in the fluorouracil-based group) and neutropenia fever–induced hospitalization also occurred in a greater percentage of patients in the paclitaxel-based group (40% vs 17% in the fluorouracil-based group). Regarding gastrointestinal toxic effects, a 2007 meta-analysis^12^ showed that cisplatin-based chemotherapy resulted in a significantly higher rate of gastrointestinal toxic effects (18% vs 8%) than that of carboplatin-based chemotherapy. In our study, a significantly higher percentage of patients in the cisplatin group experienced grade 3 or higher gastrointestinal toxic effects than those in the other 2 groups, but still a lower percentage than those in the cisplatin plus fluorouracil group in the ESO-Shanghai 1 trial (grade ≥3 vomiting, 18.7%), which might be due to the addition of aprepitant, a NK-1 receptor antagonist, together with the 5-TH3 receptor antagonists used in this trial according to the latest antiemesis guidelines. Higher percentages of patients with gastrointestinal toxic effects in the cisplatin group led to more grade 3 or higher hyponatremia events in this group (5.6% vs 0% for fluorouracil and 1.9% carboplatin).

### Limitations

Several limitations of this study should be considered. First, in view of the similarity in survival and difference in adverse events between different groups, quality of life should be added to the study, and a noninferiority study design would be more meaningful. In addition, for the radiation dose, a total dose of 61.2 Gy was delivered in this study instead of the 50.4 Gy dose that is common in Western countries. The main reason for this difference was that the standard radiation dose for radical chemoradiotherapy was still 60 to 70 Gy in Chinese guidelines and clinical practice. We believe that these differences did not alter the results of the study because patients were randomized into 3 groups that received the same radiotherapy regimen.

## Conclusions

In a comparison of paclitaxel-based chemotherapy regimens, fluorouracil did not show OS superiority over cisplatin or carboplatin in definitive chemoradiation for patients with locally advanced ESCC. Different toxicity profiles were observed. The weekly fluorouracil or carboplatin plans were accompanied by relatively mild toxic effects while the monthly cisplatin regimen led to higher rates of hematologic and gastrointestinal toxic effects.

## Notes

Supplement 1. **eTable 1.** Recruitment by Center **eTable 2.** Inclusion and Exclusion Criteria **eTable 3.** Chemotherapy Compliance in Randomly Assigned Patients **eTable 4.** Radiotherapy Parameters and Compliance in Randomly Assigned Patients **eTable 5.** Pattern of Treatment Failure **eFigure 1.** Overall Survival in N0, N1, and M1 Patients **eFigure 2.** Subgroup Analyses of the Overall Survival Between the Paclitaxel Plus Fluorouracil Group and the Paclitaxel Plus Cisplatin Group or the Paclitaxel Plus Carboplatin Group **eFigure 3.** Locoregional Recurrence-free Survival and Distant Metastasis-free Survival in Enrolled Patients

Supplement 2.
Trial Protocol

Supplement 3.
Data Sharing Statement
