# Supplementary material for: Comparison of 3 Paclitaxel-Based Chemoradiotherapy Regimens for Patients With Locally Advanced Esophageal Squamous Cell Cancer: A Randomized Clinical Trial
Source: JAMA Netw Open. 2022 Feb 21;5(2):e220120. doi: 10.1001/jamanetworkopen.2022.0120 (PMC8861838; doi:10.1001/jamanetworkopen.2022.0120)
Supplement: Supplement 1. — eTable 1. Recruitment by Center eTable 2. Inclusion and Exclusion Criteria eTable 3. Chemotherapy Compliance in Randomly Assigned Patients eTable 4. Radiotherapy Parameters and Compliance in Randomly Assigned Patients eTable 5. Pattern of Treatment Failure eFigure 1. Overall Survival in N0, N1, and M1 Patients eFigure 2. Subgroup Analyses of the Overall Survival Between the Paclitaxel Plus Fluorouracil Group and the Paclitaxel Plus Cisplatin Group or the Paclitaxel Plus Carboplatin Group eFigure 3. Locoregional Recurrence-free Survival and Distant Metastasis-free Survival in Enrolled Patients [file jamanetwopen-e220120-s001.pdf]

Supplemental Online Content

Ai D, Ye J, Wei S, et al. Comparison of 3 paclitaxel-based chemoradiotherapy regimens for patients with locally advanced esophageal squamous cell cancer: a randomized clinical trial. *JAMA Netw Open*. 2022;5(2):e220120. doi:10.1001/jamanetworkopen.2022.0120

eTable 1. Recruitment by Center

eTable 2. Inclusion and Exclusion Criteria

eTable 3. Chemotherapy Compliance in Randomly Assigned Patients

eTable 4. Radiotherapy Parameters and Compliance in Randomly Assigned Patients

eTable 5. Pattern of Treatment Failure

eFigure 1. Overall Survival in N0, N1, and M1 Patients

eFigure 2. Subgroup Analyses of the Overall Survival Between the Paclitaxel Plus Fluorouracil Group and the Paclitaxel Plus Cisplatin Group or the Paclitaxel Plus Carboplatin Group

eFigure 3. Locoregional Recurrence-free Survival and Distant Metastasis-free Survival in Enrolled Patients

This supplemental material has been provided by the authors to give readers additional information about their work.

© 2022 Ai D et al. *JAMA Network Open.*

eTable 1. Recruitment by center

Center	Principle Investigator	No. patients
Fudan University Shanghai Cancer Center	Kuaile Zhao	232
Jiangsu Province Cancer Hospital	Jinjun Ye	57
Gansu Province Cancer Hospital	Shihong Wei	12
Fudan University Shanghai Cancer Center Minhang Branch	Yunhai Li	6
Jiangxi Province Cancer Hospital	Hui Luo	5
Shanxi Province Cancer Hospital	Jianzhong Cao	4
The First Affiliated Hospital of Xiamen University	Qin Lin	2
Huadong Hospital Affiliated to Fudan University	Xiangpeng Zheng	1
Affiliated Hospital of Jiangnan University	Jialiang Zhou	1
Hainan Province People’s Hospital	Guang Huang	1
Fujian Province Cancer Hospital	Jiancheng Li	0

© 2022 Ai D et al. *JAMA Network Open.*

eTable 2. Inclusion and exclusion criteria

Inclusion Criteria	Exclusion Criteria
Histologically confirmed esophageal squamous cell carcinoma.	Esophageal perforation or hematemesis
Clinical stages II, III or IVa based on the sixth American Joint Committee on Cancer tumor, node, metastasis classification	Received thoracic, abdominal or craniocerebral surgery within 30 days
No prior treatments of chemotherapy, radiotherapy or surgery against esophageal cancer, except for non-curative resection by endoscopic mucosal resection/endoscopic sub mucosal dissection	Synchronous or metachronous malignancies (except for cutaneous [non-melanoma] carcinoma, thyroid papillary carcinoma, phase I seminoma or cervical carcinoma in situ curatively treated and disease free for a minimum of 3 months)
Aged 18–75 years	Enrolled in other clinical trials within 30 days
Adequate organ functions for chemoradiation therapy:<br>(1) White blood cell count ≥3×10⁹⁄L;<br>(2) Absolute neutrophil counts ≥1.5×10⁹⁄L;<br>(3) Hemoglobin ≥10g⁄dL;<br>(4) Platelet ≥100×10⁹⁄L;<br>(5) Total bilirubin <1.5upper limit of normal (ULN);<br>(6) Aspartate transaminase ≤2.5 ULN;<br>(7) Alanine aminotransferase ≤2.5 ULN;<br>(8) Creatinine ≤1.5 ULN	Unstable angina and/or congestive heart failure requiring hospitalization within 6 months

© 2022 Ai D et al. *JAMA Network Open.*

Eastern Cooperative Oncology Group performance status of 0–2<br>Life expectancy ≥3 months, based on the judgment of doctors	Severe psychiatric disease
Written informed consent	Pregnancy, lactation or unwillingness to adopt contraception
	Drug addiction
	AIDS based on current Centers for Disease Control and Prevention definition
	History of radiotherapy in the planning area
	Other ineligible conditions according to researchers

© 2022 Ai D et al. *JAMA Network Open.*

eTable 3. Chemotherapy compliance in randomly assigned patients

Chemotherapy Compliance	Paclitaxel Plus Fluorouracil Group No. (%) (n=107)	Paclitaxel Plus Cisplatin Group No. (%) (n=107)	Paclitaxel Plus Carboplatin Group No. (%) (n=107)
Concurrent cycles			
1	2 (1.9)	13 (12.1)	1 (0.9)
2	8 (7.5)	94 (87.9)	2 (1.9)
3	2 (1.9)	-	9 (8.4)
4	14 (13.1)	-	10 (9.3)
5	22 (20.6)	-	17 (15.9)
6	59 (55.1)	-	68 (63.6)
Completed over 80%	81 (75.7)	94 (87.9)	85 (79.4)
Consolidation cycles			
0	35 (32.7)	35 (32.7)	32 (29.9)
1	11 (10.3)	17 (15.9)	12 (11.2)
2	61 (57.0)	55 (51.4)	63 (58.9)
Deduction in dose			
Yes	6 (5.6)	38 (35.5)	9 (8.4)
No	101 (94.4)	69 (64.5)	98 (91.6)
Delay in cycles			
Yes	65 (60.7)	51 (47.7)	67 (62.6)
No	42 (39.3)	56 (52.3)	40 (37.4)

© 2022 Ai D et al. *JAMA Network Open.*

eTable 4 Radiotherapy parameters and compliance in randomly assigned patients

Radiotherapy Parameters and Compliance	Paclitaxel Plus Fluorouracil Group No. (%) (n=107)	Paclitaxel Plus Cisplatin Group No. (%) (n=107)	Paclitaxel Plus Carboplatin Group No. (%) (n=107)
Finished 61.2Gy	100 (93.5)	102 (95.3)	96 (89.7)
Median dose for unfinished, Gy (Range)	50.4 (10.8-59.4)	55.8 (41.4-59.4)	57.6 (7.2-59.4)
Interruption in radiotherapy			
No Interruption	82 (76.6)	66 (61.7)	79 (73.8)
Interruption during radiotherapy	25 (23.4)	41 (38.3)	28 (26.2)

© 2022 Ai D et al. *JAMA Network Open.*

eTable 5. Pattern of treatment failure

Type of Event	Paclitaxel Plus Fluorouracil Group No. (%) (n=107)	Paclitaxel Plus Cisplatin Group No. (%) (n=107)	Paclitaxel Plus Carboplatin Group No. (%) (n=107)
Live without treatment failure	51 (47.6)	48 (44.9)	47 (43.9)
Failure	56 (52.3)	59 (55.1)	60 (56.1)
Locoregional only	26 (24.3)	27 (25.2)	24 (22.4)
Distant only	11 (10.3)	17 (15.9)	22 (20.6)
Locoregional and distant	5 (4.7)	7 (6.5)	8 (7.5)
Second primary cancer	8 (7.5)	5 (4.7)	3 (2.8)
Toxicity-induced death	2 (1.9)	0	1 (3.7)
Death of other course[a]	4 (3.7)	3 (2.8)	2 (1.9)

[a] One patient in each of the TF, TP and TC group died as a result of pneumonia, one patient in the TP group died of pulmonary embolism, one patient in the TF group died of postoperative complications, one patient in the TC group died of myocardial infarction, and two patients in the TF group and one patient in the TP group died of unknown causes.

© 2022 Ai D et al. *JAMA Network Open.*

eFigure 1. Overall survival in N0 (A), N1 (B), and M1 (C) patients

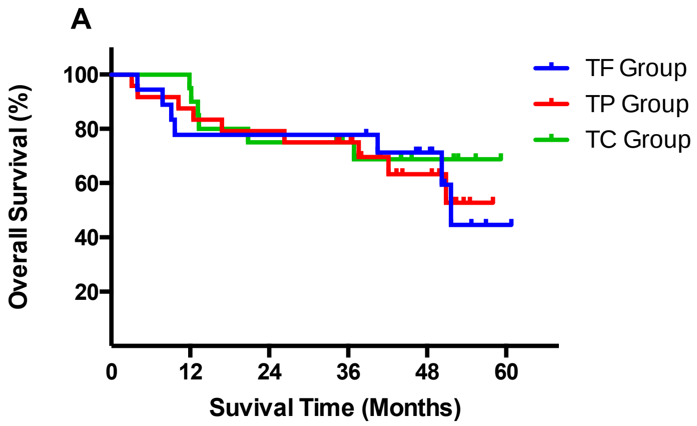

No. at risk						
TF group	18	14	14	14	9	1
TP group	24	21	19	16	8	0
TC group	20	19	15	12	5	0

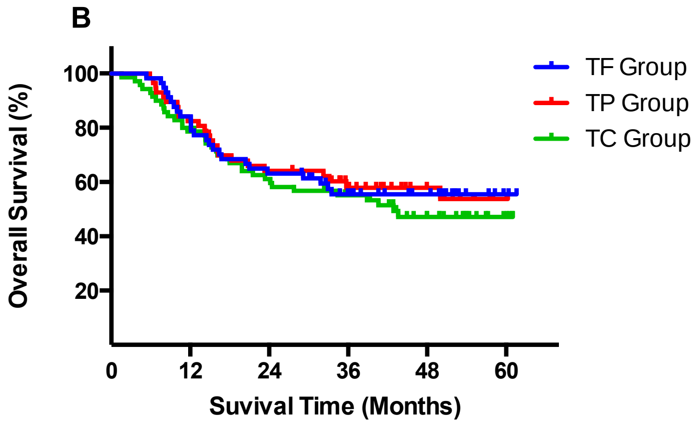

No. at risk						
TF group	57	48	36	25	17	2
TP group	57	47	34	25	14	3
TC group	70	55	42	31	18	4

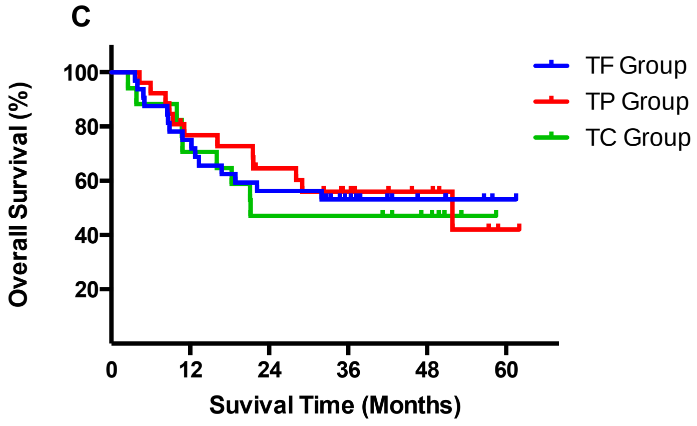

No. at risk						
TF group	32	24	18	13	6	1
TP group	26	19	15	10	6	1
TC group	17	12	8	8	5	0

Paclitaxel/fluorouracil did not show superiority over paclitaxel/cisplatin or paclitaxel/carboplatin regimen in terms of overall survival within different stage subgroups.

© 2022 Ai D et al. *JAMA Network Open.*

eFigure 2. Subgroup analyses of the overall survival between the paclitaxel plus fluorouracil group and the paclitaxel plus cisplatin group (A) or the paclitaxel plus carboplatin group (B)

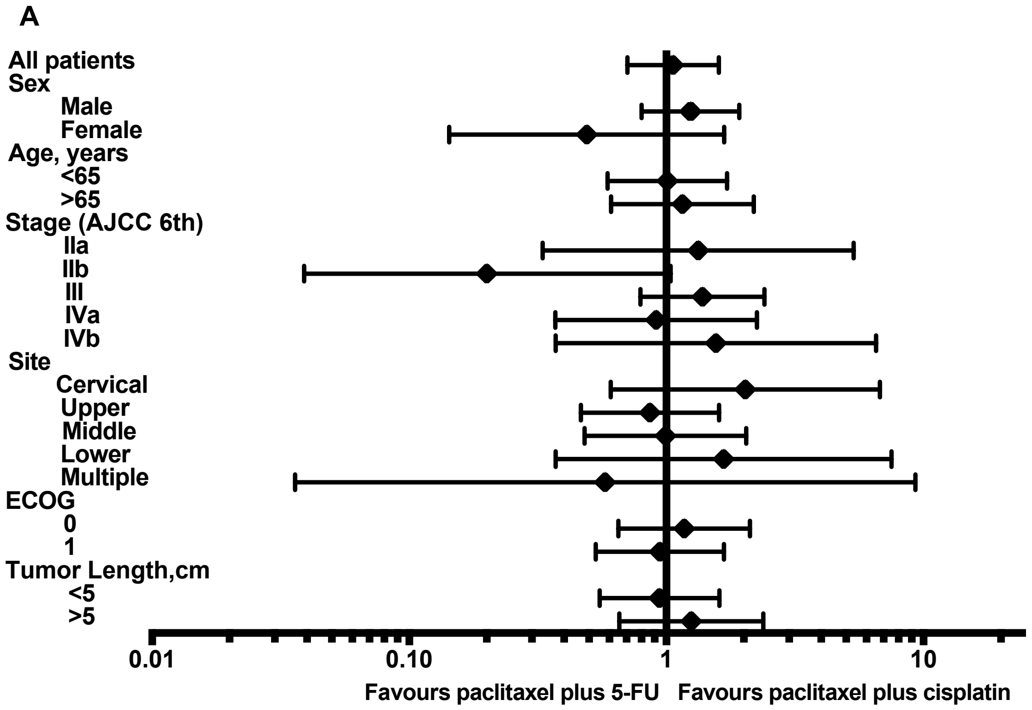

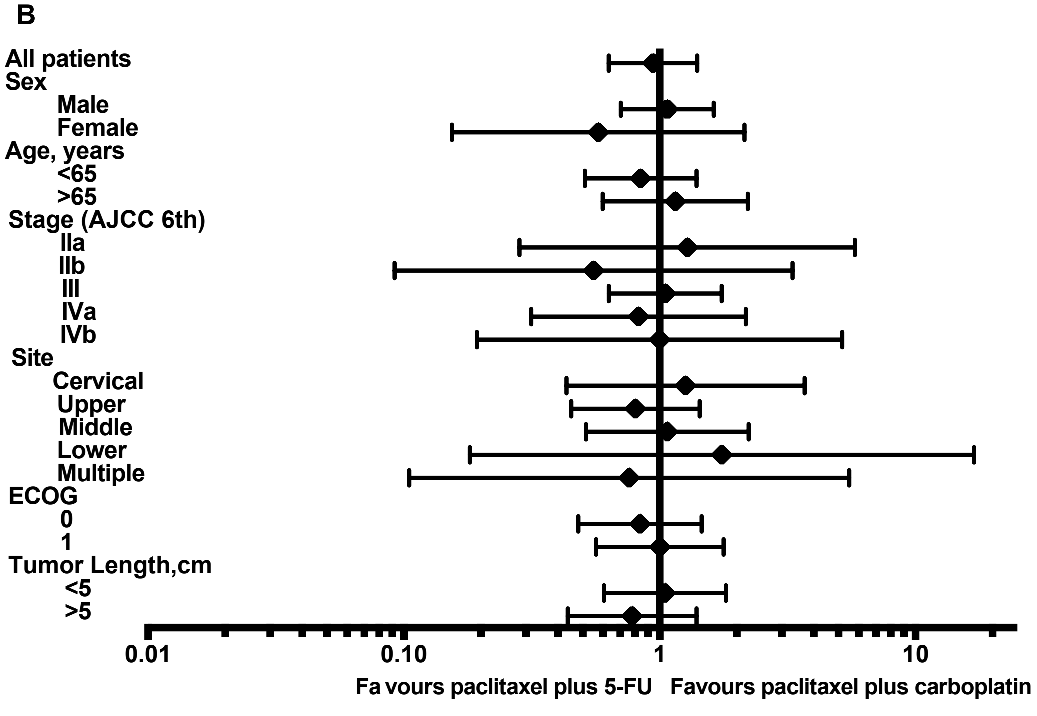

The effects of different regimens on the overall survival according to the predictive and prognostic factors (sex, age, smoking history, stage, tumor site, ECOG, and tumor length) were not significantly different between the two groups. Abbreviations: AJCC, American Joint Committee on Cancer; ECOG, Eastern Cooperative Oncology Group.

© 2022 Ai D et al. *JAMA Network Open.*

eFigure 3. Locoregional recurrence-free survival (A) and distant metastasis-free survival (B) in enrolled patients

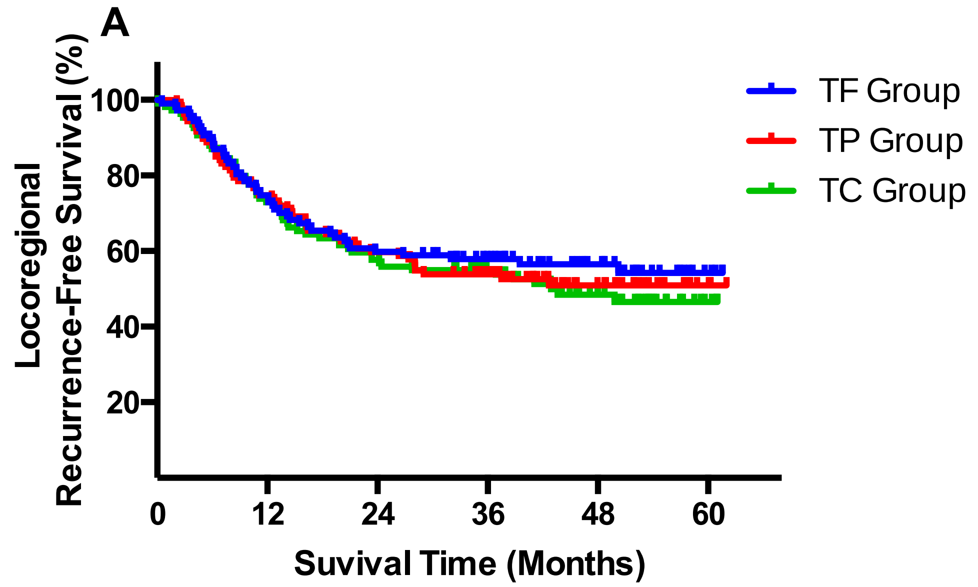

No. at risk						
TF group	107	80	64	51	30	4
TP group	107	80	62	46	24	4
TC group	107	78	61	48	27	3

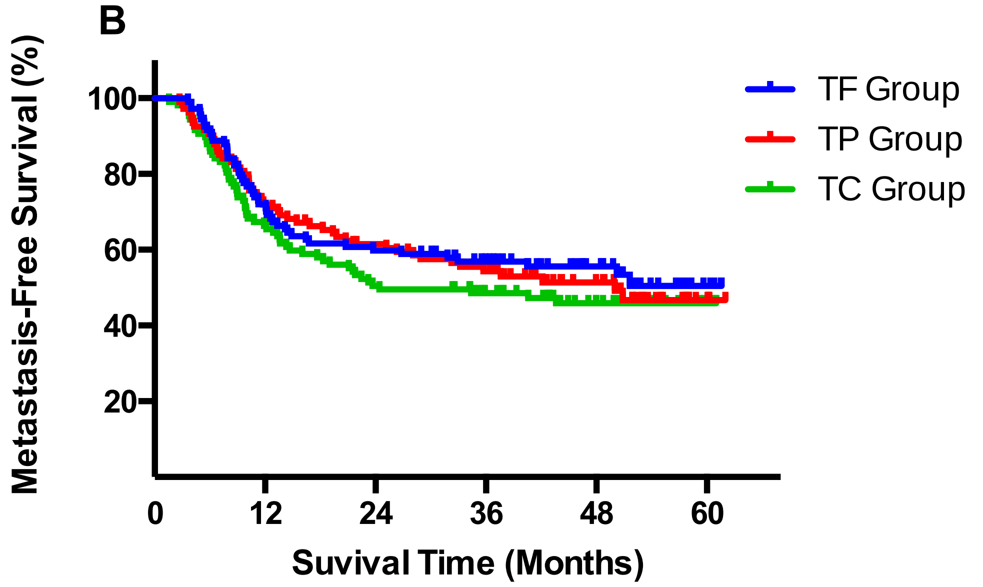

No. at risk						
TF group	107	77	64	51	31	4
TP group	107	76	63	45	26	4
TC group	107	71	54	43	26	4

Paclitaxel/fluorouracil did not show superiority over paclitaxel/cisplatin or paclitaxel/carboplatin regimen in terms of locoregional recurrence-free survival or distant metastasis-free survival.

© 2022 Ai D et al. *JAMA Network Open.*
