# Supplementary material for: Comparison of 3 Paclitaxel-Based Chemoradiotherapy Regimens for Patients With Locally Advanced Esophageal Squamous Cell Cancer: A Randomized Clinical Trial
Source: JAMA Netw Open. 2022 Feb 21;5(2):e220120. doi: 10.1001/jamanetworkopen.2022.0120 (PMC8861838; doi:10.1001/jamanetworkopen.2022.0120)
Supplement: Supplement 2. — Trial Protocol [file jamanetwopen-e220120-s002.pdf]

Ai D, Ye J, Wei S, et al. Comparison of 3 paclitaxel-based chemoradiotherapy regimens for patients with locally advanced esophageal squamous cell cancer: a randomized clinical trial. *JAMA Netw Open*. 2022;5(2):e220120. doi:10.1001/jamanetworkopen.2022.0120

Protocol (English Version)

Comparison of paclitaxel in combination with cisplatin (TP), carboplatin (TC) or fluorouracil (TF) concurrent with radiotherapy for patients with local advanced esophageal squamous cell carcinoma: a three-arm phase III randomized trial (ESO-Shanghai 2)

Approved by Fudan University Shanghai Cancer Center Institutional Review Board (Ethics Committee of Fudan University Shanghai Cancer Center：No.1505146-13)

List of abbreviations:

TP: Paclitaxel combined with cisplatin

TC: Paclitaxel combined with carboplatin

TF: Paclitaxel combined with fluorouracil

UICC: Union for International Cancer Control

ESCC: Esophageal squamous cell carcinoma

PF: Cisplatin combined with fluorouracil

AIDS: Acquired immune deficiency syndrome

RT: Radiotherapy

PTX: Paclitaxel

DDP: Cisplatin

CBP: Carboplatin

5-FU: Fluorouracil

W: Week

ICRU: International Commission on Radiation Units and Measurements

GTV: Gross Target Volume

CTV: Clinical Target Volume

PTV: Planning Target Volume

WBC: White Blood Cell

ANC: Absolute Neutrophil Counts

Hb: Hemoglobin

Plt: Platelet

ULN: Upper Limit of Normal

AST: Aspartate Transaminase

ALT: Alanine aminotransferase

1. Introduction

Worldwide, esophageal cancer is the eighth most common cancer, which is responsible for an estimated 455,800 new cases and 400,200 deaths in 2012.[1] Since its prognosis is dismal, much effort has been put into improving overall survival through multi-modality treatments, which consist of surgery, radiotherapy and chemotherapy.[2] Concurrent chemoradiation is the standard non-operative therapy for local advanced esophageal squamous cell carcinoma (ESCC).[3]

Paclitaxel is an active agent against esophageal cancer, with the response rate of 28% in ESCC, and it has been shown to be a potent radiation sensitizer.[4] There have been multiple studies evaluating paclitaxel-based chemoradiation in esophageal cancer, for instance, paclitaxel/fluorouracil (TF) developed at The University of Texas M.D. Anderson Cancer Center, and paclitaxel/cisplatin (TP) developed at Memorial Sloan-Kettering Cancer Center,[5 6] with paclitaxel/carboplatin (TC) from CROSS trial.[7] In many preoperative studies, paclitaxel-based chemoradiotherapy has achieved inspiring effect, the pathologic complete response rates of TP-based chemoradiotherapy were 19%-42%, [8-11] and of TC-based chemoradiotherapy was 49%.[7] However, which regimen, among TF, TP and TC-based definitive chemoradiotherapy, provides best prognosis with minimum adverse events is still considered far from resolved and very few studies focus on this field.

RTOG 0113[5] evaluated 2 different paclitaxel-based regimens (TP and TF). Eighty-four patients were accrued to this study. Patients in arm A (TF) received induction 5-FU, cisplatin, and paclitaxel followed by radiation and concurrent continuous infusion 5-FU and weekly paclitaxel. Patients in arm B (TP) received induction paclitaxel and cisplatin followed by radiation and concurrent weekly cisplatin and 96-hour infusion of paclitaxel. The median survival time was 28.7 months for patients in arm A (TF) and 14.9 months for patients in arm B (TP). Neither arm achieved the hypothesized 1-year survival rate of at least 77.5%. The main deficiency of this study is the small sample size, but the effect of TF group is still inspiring.

Another retrospective study from Europe[12] showed the overall survival of TC-based definitive chemoradiotherapy was comparable with cisplatin/5-FU (PF) as definitive concurrent chemoradiotherapy in esophageal cancer. However, the toxicity rates were lower in the TC group together with higher treatment compliance.

Reference:

1. Torre LA, Bray F, Siegel RL, et al. Global Cancer Statistics, 2012. Ca-Cancer J Clin 2015;**65**(2):87-108.
2. Enzinger PC, Mayer RJ. Esophageal cancer. N Engl J Med 2003;**349**(23):2241-52.
3. Herskovic A, Martz K, Alsarraf M, et al. Combined Chemotherapy and Radiotherapy Compared with Radiotherapy Alone in Patients with Cancer of the Esophagus. New Engl J Med 1992;**326**(24):1593-98.
4. Ajani JA, Ilson DH, Daugherty K, et al. Activity of Taxol in Patients with Squamous-Cell Carcinoma and Adenocarcinoma of the Esophagus. J Natl Cancer I 1994;**86**(14):1086-91.
5. Ajani JA, Winter K, Komaki R, et al. Phase II randomized trial of two nonoperative regimens of induction chemotherapy followed by chemoradiation in patients with localized carcinoma of the esophagus: RTOG 0113. J Clin Oncol 2008;**26**(28):4551-56.
6. Schnirer II, Komaki R, Yao JC, et al. Pilot study of concurrent 5-fluorouracil/paclitaxel plus radiotherapy in patients with carcinoma of the esophagus and gastroesophageal junction. Am J Clin Oncol-Canc 2001;**24**(1):91-95.
7. Shapiro J, Van Lanschot JJB, Hulshof MCCM, et al. Neoadjuvant chemoradiotherapy plus surgery versus surgery alone for oesophageal or junctional cancer (CROSS): long-term results of a randomised controlled trial. Lancet Oncol 2015;**16**(9):1090-98.
8. Safran H, Gaissert H, Akerman P, et al. Paclitaxel, cisplatin, and concurrent radiation for esophageal cancer. Cancer Invest 2001;**19**(1):1-7.
9. Bains MS, Stojadinovic A, Minsky B, et al. A phase II trial of preoperative combined-modality therapy for localized esophageal carcinoma: Initial results. J Thorac Cardiov Sur 2002;**124**(2):270-77.
10. Urba SG, Orringer MB, Ianettonni M, et al. Concurrent cisplatin, paclitaxel, and radiotherapy as preoperative treatment for patients with locoregional esophageal carcinoma. Cancer 2003;**98**(10):2177-83.
11. Lin CC, Hsu CH, Cheng JC, et al. Concurrent chemoradiotherapy with twice weekly paclitaxel and cisplatin followed by esophagectomy for locally advanced

esophageal cancer. Ann Oncol 2007;**18**(1):93-98.
12. Honing J, Smit JK, Muijs CT, et al. A comparison of carboplatin and paclitaxel with cisplatinum and 5-fluorouracil in definitive chemoradiation in esophageal cancer patients. Ann Oncol 2014;**25**(3):638-43.

2. Study objectives

2.1 Primary objective

- To assess the overall survival of TF compared with TP
- To assess the overall survival of TF compared with TC

2.2 Secondary objective

- To assess the progression-free survival of TF compared with TP or TC
- To assess the safety and tolerability profile of TF compared with TP or TC

3. Study plan and procedures

3.1 Overall study design

The trial is a three-arm, multicenter, open-labeled, randomized phase III clinical trial to confirm the priority of TF to TP and TF to TC concurrent with definitive radiotherapy in terms of overall survival for patients with local advanced esophageal squamous cell carcinoma.

A total of 321 patients with local advanced esophageal squamous cell carcinoma will be randomized in a 1:1:1 ratio to 1 of 3 arms:

- Paclitaxel+5-Fu (TF)
- Paclitaxel+cisplatin (TP)
- Paclitaxel+carboplatin (TC)

Randomization will be stratified by lymph node status: N0, N1, M1a, according to UICC 6th TNM classification

Patients will receive radiotherapy combined with concurrent chemotherapy. Radiotherapy will commence on Day 1 following randomization, concurrent with the beginning of cycle 1 of chemotherapy.

The study design is shown in Figure 1 and Table 1.

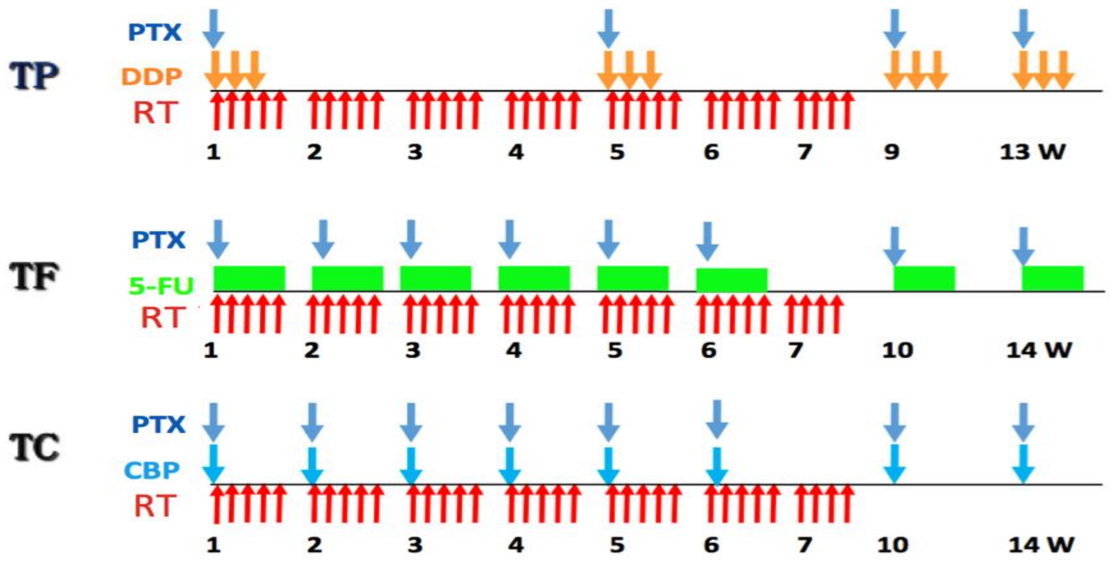

Figure 1. Study design of the ESO-Shanghai 2 trial.

Table1. Schedule of study procedures

Evaluation	Screening	Treatment												Follow up
	（≤ -2w）	1	2	3	4	5	6	7	8	9	11	13	15	Year 1 and 2: Once per 3 months<br>Year 3-5: Once per 6 months<br>From Year 6: Once per year
Written informed consent	√													
Verify eligibility criteria	√													
Randomization	√													
Physical examination	√							√		√			√	√
Electrocardiogram	√							√		√			√	√
WHO performance status	√							√		√			√	√
Endoscopy/biopsy	√													
Hematology	√	√	√	√	√	√	√	√	√	√	√	√	√	√
Serum chemistry	√				√			√		√		√	√	√
Chest CT with contrast	√												√	√
Barium swallow	√			√		√							√	√

Abdomen CT/MRI/ultrasound	√							√					√	√
AE assessment	√	√	√	√	√	√	√	√	√	√	√	√	√	√

4. Patient selection

4.1 Inclusion criteria

1. Histologically confirmed esophageal squamous cell carcinoma
2. Clinical stages II, III or IVa based on the 6th UICC-TNM classification
3. No prior treatment of chemotherapy, radiotherapy or surgery against esophageal cancer, except for non-curative resection by EMR/ESD.
4. Aged 18-75 years
5. Adequate organ functions for chemoradiation therapy
   a) White blood cell (WBC) $\geq 3\times10^9$/L
   b) Absolute neutrophil counts (ANC) $\geq 1.5\times10^9$/L
   c) Hemoglobin (Hb) ≥10g/dl
   d) Platelet (Plt) $\geq 100\times10^9$/L
   e) Total bilirubin <1.5 upper limit of normal (ULN)
   f) Aspartate transaminase (AST) ≤2.5 ULN
   g) Alanine aminotransferase (ALT) ≤2.5 ULN
   h) Creatinine ≤1.5 ULN
6. ECOG PS of 0-2
7. Life expectancy ≥3 months
8. Written informed consent

4.2 Exclusion criteria

1. Esophageal perforation or hematemesis
2. Synchronous or metachronous malignancies (except for cutaneous (non-melanomas) carcinoma, thyroid papillary carcinoma, phase I seminoma or cervical carcinoma in situ curatively treated and disease free for a minimum of 3 months)
3. Received thoracic, abdominal or craniocerebral surgery within 30 days
4. Enrolled in other clinical trials within 30 days
5. Unstable angina and/or congestive heart failure requiring hospitalization within 6 months
6. Severe psychiatric disease
7. Pregnancy, lactation or unwillingness to adopt contraception

8. Drug addiction
9. Acquired immune deficiency syndrome (AIDS) based upon current CDC definition
10. History of radiotherapy in the planning area
11. Other ineligible conditions according to researchers

5. Patient enrollment and randomization

After the confirmation of eligibility criteria, patients will be randomly allocated in a 1:1:1 ratio to the three treatment groups by a central randomization center (Fudan University Shanghai Cancer Center, Shanghai, China). Patients will be stratified by lymph node status (N0, N1, M1a). The SAS will be used to generate a random permutation sequence and produce patient randomization numbers. After receiving the information of the patients, the data center will register the enrollment, assign a unique identification number to every participant, and reply to the respective investigators with the result of randomization. If a patient discontinues participation in the study, then their enrollment/randomization number cannot be reused.

No blinding exists in this study.

6. Treatment

The treatment plan is shown in Figure 1. Patients receive radiotherapy combined with concurrent chemotherapy. Radiotherapy begins on day 1, concurrent with the beginning of cycle 1 of chemotherapy.

6.1 Radiation therapy

Same radiation therapy will be delivered in all three treatment groups. Radiotherapy is delivered with photons (≥6 MV) to a total dose of 61.2Gy in 34 fractions. Patients will be treated 5 days per week at 1.8Gy/d. Three-dimensional conformal radiotherapy or intensity modulated radiotherapy is required. All patient will be positioned in an individualized immobilization device in the treatment position.

The definition of volumes will be in accordance with the 1993 ICRU Report #50 and 1999 ICRU Report #62.

The gross target volume (GTV) is defined as all known involved field, which detected by endoscopic ultrasound, barium swallow or CT scan (whichever is larger).

The regional lymph nodes included in GTV is whose diameter more than 1cm (0.5cm for lymph nodes at tracheoesophageal groove) or histologically proven metastatic after puncture.

The superior and inferior borders of the clinical target volume (CTV) are 3cm beyond the primary tumor along the esophagus. The lateral, anterior and posterior borders of the field are the same as GTV.

The superior, inferior, anterior, posterior and lateral borders of planning target volume (PTV) are 1cm beyond CTV. Field next to the spinal cord could be slightly adjusted in order to reduce the exposure of spinal cord.

As for target volume, tissue inhomogeneity correction is adopted and it is required that more than 99% PTV receive 95% prescription dose and more than 95% PTV receive 99% or more prescription dose. Highest and lowest point dose inside PTV should be recorded.

When making the treatment plan, we should take normal organ dose restrictions into consideration as the following order: (Table 2)

Risk organ	Contour regulation	Dose restriction
Spinal cord	All the layers of CT scan have to be contoured and the margin of vertebra tube can be regarded as that of planning organ at risk volume.	Highest point dose less than 45Gy
Lung	It is allowed to use automatic tools in the delineation of margin of lungs. (Trachea and bronchia must be contoured manually)	The volume of lung (PTV excluded) receiving 20Gy or higher has to be less than 30% of the total lung volume, and the mean dose has to be less than 15Gy.
Heart	The superior margin of heart consists of right atrium and right ventricle, pulmonary	The mean dose has to be less than 40Gy.

	artery trunk, ascending main aorta and superior vena cava excluded. The inferior margin is at the level of heart apex.	

Table 2. Contour regulation and dose restriction of risk organs

6.2 Chemotherapy

Patients are randomly assigned to receive one of three therapies.

- Arm A (TP)

Patients in arm A will receive 4 courses of TP every 4 weeks. Details are as follows:

Paclitaxel: 175mg/m^2/d, ivgtt over 3 hours, d1; Cisplatin: 25mg/m^2/d, ivgtt, d1-3;

- Arm B (TF)

Patients in arm B will receive 6 courses of TF concurrent with radiotherapy every week and 2 courses of TF consolidation chemotherapy every 4 weeks. Details are as follows:

Concurrent: paclitaxel 50mg/m^2/d, ivgtt over 3 hours, d1; 5-FU 300mg/m^2, civ 96h, d1-4

Consolidation: paclitaxel 175 mg/m^2/d, ivgtt over 3 hours, d1; 5-FU 1800mg/m^2, civ 72h, d1-3

- Arm C (TC)

Patients in arm C will receive 6 courses of TC concurrent with radiotherapy every week and 2 courses of TC consolidation chemotherapy every 4 weeks. Details are as follows:

Concurrent: paclitaxel 50mg/m^2/d, ivgtt over 3 hours, d1; carboplatin AUC=2, ivgtt, d1

Consolidation: paclitaxel 175 mg/m^2/d, ivgtt over 3 hours, d1; carboplatin AUC=5, ivgtt, d1

Patients will receive premedication to prevent allergic reaction and significant nausea or vomiting as indicated.

6.3 Dose modifications

6.3.1 Radiotherapy interruption

If following toxicity is observed, radiotherapy has to be delayed until toxicity is no more than grade 2.

- WBC$<2.0\times10^{9}$/L or ANC$<1.0\times10^{9}$/L
- Plt$<50\times10^{9}$/L
- Grade 3 or higher non-hematological toxicity

If following toxicity is observed, radiotherapy has to be delayed until complete recovery.

- Mediastinal or thoracic infection with fever over 38.5℃

It is allowed to suspend at most 2 weeks, or radiotherapy will be terminated.

6.3.2 Chemotherapy interruption and dose modifications

If following toxicity is observed on day 1, chemotherapy has to be delayed until toxicity is no more than grade 1.

- ANC$<1.5\times10^{9}$/L
- Plt$<100\times10^{9}$/L
- Grade 2 or higher non-hematological toxicity, except for nausea, vomiting and alopecia

It is allowed to delay at most 2 weeks, or chemotherapy will be terminated.

Chemotherapy dose modifications are based on the greatest toxicity during the last cycle. Any patients who need to make chemotherapy dose modifications will receive the modified dose in the following cycles.

If modifications are needed, dose of paclitaxel, cisplatin, carboplatin and 5-FU will decreased by 25% from the planned dose for the first time and 50% for the second time. It is allowed to make dose modifications at most twice, or chemotherapy will be terminated. Details are as follows:

Dose modification of paclitaxel

- Febrile neutropenia (ANC$<$ 0.5×10^{9}/L and fever over 38.3℃ or over 38.0℃ for 1h)
- Grade 2 or higher peripheral neuropathy

Dose modification of cisplatin and carboplatin

- Febrile neutropenia (ANC$<$ 0.5×10^{9}/L and fever over 38.3℃ or over 38.0℃ for 1h)
- Grade 2 or higher peripheral neuropathy
- Serum creatinine $>$3ULN

Dose modification of 5-FU

- Febrile neutropenia (ANC$<$ 0.5×10^{9}/L and fever over 38.3℃ or over 38.0℃ for 1h)

- Grade 3 or higher mucositis

7. Data collection

7.1 Data collection at enrollment

The following assessments and procedures should be performed within 14 days prior to randomization.

- Written informed consent for the study and assignment of a patient identification number
- Verify eligibility criteria
- Physical examination to assess all conditions that are current and ongoing
- WHO performance status
- Hematology, clinical chemistry
- 12-lead ECG recording
- Chest CT with contrast, Barium swallow, and Abdomen CT/MRI/ultrasound

7.2 Data collection at follow-up

Participants will be seen at hospital or contacted by telephone and letters from randomization to the end of treatment cycle, then at Month 3, 6, 9, 12, 15, 18, 21, 24, 30, 36, 42, 48, 54 and 60 after last treatment. Any disease and survival status and adverse events must be followed-up and noted in CRFs. Chest CT scan with contrast, abdomen CT/MRI/ultrasound and barium swallow should be processed as routine and endoscopy when necessary. Investigators will review all the results of the examinations above and make disease assessment.

7.3 Efficacy and safety data collection

The primary endpoint is overall survival in all randomized patients. Overall survival is defined as time from the date of randomization until death. The secondary endpoint is progression free survival (PFS) and adverse events. PFS is defined as the time from the date of randomization to the date of progression or to the date of death, whichever occurs first and disease progression will be evaluated according to RECIST Version 1.1. The adverse events will be evaluated according to the National Cancer Institute Common Terminology Criteria for Adverse Events (CTCAE version

4.0). All adverse events, occurring during the course of the trial, which is from randomization until 28 days after end of treatment, regardless of relatedness to study medication, will be recorded. Adverse events occurring later than 28 days after the end of treatment will only be recorded if they are considered relevant.

8. Statistical methods and sample size determination

8.1 Description of analysis sets

The statistical analysis of overall survival, progression-free survival will include all randomized patients and will compare the treatment arms on the basis of randomized treatment, regardless of the treatment actually received.

All patients who received at least one cycle of randomized study drug will be included in the safety population. Throughout the safety results sections, erroneously treated patients (eg, those randomized to Treatment A but actually given Treatment B) will be accounted for in the actual treatment arm.

8.2 Methods of statistical analyses

The median OS and PFS will be estimated with Kaplan-Meier method and log-rank test will be used to compare the overall survival among treatment arms. Cox regression analysis was performed to estimate the hazard ratios of overall survival of TF vs. TP or TF vs. TC group with a 97.5% confidence interval (CI).

Adverse events will be listed individually by patient. The number of patients experiencing each AE will be summarized by treatment arm and CTCAE grade.

8.3 Determination of the sample size

The sample size for this study was selected to be consistent with the research hypothesis.

According to the RTOG 0113 study and other retrospective reports, the median survival time of patients with ESCC receiving TF concurrent with radiotherapy is 28.7 months, compared with 14.9 months for TP and 17.4 months for TC, which will be used for sample size determination.

There were two pairs of comparisons:

- Comparison 1: Paclitaxel plus 5-Fu (TF) vs. paclitaxel plus cisplatin (TP)
- Comparison 2: Paclitaxel plus 5-Fu (TF) vs. Paclitaxel plus carboplatin (TC)

To control for type-I error, a significance level of 2.5% will be used for analysis of Comparison 1 and a significance level of 2.5% will be used for analysis of

Comparison 2. The study will be considered positive (a success) if either analysis results are statistically significant.

For Comparison 1, a minimum of 59 patients per arm will be necessary to warrant a power of 80% at a two-sided α level of 0.025 to demonstrate the superiority of TF to TP group, assuming an accrual period of 48 months, a minimum follow-up period of 24 months.

For Comparison 2, a minimum of 102 patients per arm will be necessary to warrant a power of 80% at a two-sided α level of 0.025 to demonstrate the superiority of TF to TC group, assuming an accrual period of 48 months, a minimum follow-up period of 24 months.

The larger sample size for single group (102 patients) within two comparisons will be chosen and therefore total sample size was 321, with 5% dropout rate.

9. Ethics and dissemination

The study will be performed in accordance with ethical principles that have their origin in the Declaration of Helsinki and are consistent with Good Clinical Practice (GCP).

This trial has been approved by all participating centers including Fudan University Shanghai Cancer Center Institutional Review Board (Ethics Committee of Fudan University Shanghai Cancer Center: No.1505146-13). Written informed consent will be obtained from all participants. Serious adverse events will be reported to the safety desk of the trial, the Data and Safety Monitoring Board and trial sites.
