# Supplementary material for: Comparison of 3 Paclitaxel-Based Chemoradiotherapy Regimens for Patients With Locally Advanced Esophageal Squamous Cell Cancer: A Randomized Clinical Trial
Source: JAMA Netw Open. 2022 Feb 21;5(2):e220120. doi: 10.1001/jamanetworkopen.2022.0120 (PMC8861838; doi:10.1001/jamanetworkopen.2022.0120)
Supplement: Supplement 3. — Data Sharing Statement [file jamanetwopen-e220120-s003.pdf]

Ai D, Ye J, Wei S, et al. Comparison of 3 paclitaxel-based chemoradiotherapy regimens for patients with locally advanced esophageal squamous cell cancer: a randomized clinical trial. *JAMA Netw Open*. 2022;5(2):e220120. doi:10.1001/jamanetworkopen.2022.0120

Data Sharing Statement

Data
Data available: No
